# Evaluating Batch Correction Methods for Large-Scale Mass Spectrometry Imaging of Heterogeneous Tissues

**DOI:** 10.1021/acs.analchem.5c04371

**Published:** 2026-01-26

**Authors:** Martin Metodiev, Alex Dexter, Weiwei Zhou, Ariadna González-Fernández, Chelsea Nikula, Lucy M. Johns, Evdoxia Karali, Emine Kazanc, Athanasios Tsalikis, Aurelien Tripp, Zoltan Takats, George Poulogiannis, Josephine Bunch

**Affiliations:** 1 9917 National Physical Laboratory, Teddington TW11 0LW, U.K.; 2 MRC Toxicology Unit, 2152 University of Cambridge, Gleeson Building, Tennis Court Road, Cambridge CB2 1QR, U.K.; 3 5053 The Institute of Cancer Research, London SW3 6JB, U.K.; 4 220091 Boğaziçi University, Istanbul 34342, Türkiye; 5 4615 Imperial College London, London W12 0NN, U.K.

## Abstract

In mass spectrometry imaging (MSI), the fluctuation in detected ion intensities, which is associated with “technical factors” and not the variability of molecular composition of the sample itself, may be referred to as “batch effects”. These batch effects are a major barrier to the more widespread uptake and use of MSI for larger clinical and preclinical studies. In other fields, such as metabolomics and transcriptomics, batch correction methods have been introduced and commonly adopted. These methods aim to mitigate systematic biases introduced by differences in experimental conditions, instruments, or processing batches in high-dimensional data, such as omics or imaging data sets. Mass spectrometry imaging poses additional challenges compared to these fields such as the need to ensure that expected intensity fluctuations throughout a sample, associated with expected spatial variability, are maintained and the inability to randomly introduce quality control spectra. To date, there is no widely adopted approach to the batch correction of mass spectrometry imaging data. In this work, we consider both stabilization of intensity variability and the usefulness of correction methods for spatially resolved data. We present a pixel-by-pixel evaluation of batch correction for mass spectrometry imaging data.

## Graphical abstract

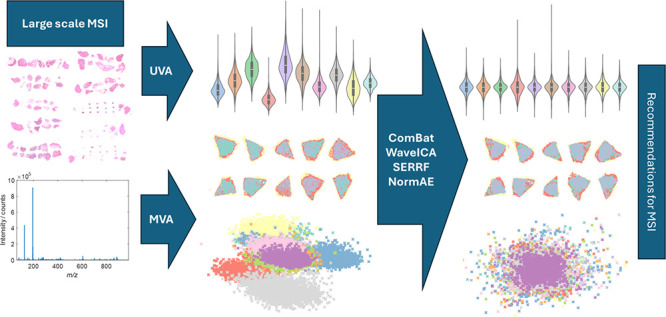

## Introduction

Mass spectrometry imaging (MSI) is a probe-based technique that provides spatially resolved, highly multiplexed information on the chemical composition of surfaces.^1^ In MSI, a surface is sampled point-by-point in a raster pattern. Maps of the chemical composition, measured as desorbed ions at every location, are generated from these data. Surface analysis of biological samples by MSI is typically carried out by matrix-assisted laser desorption ionization (MALDI) and desorption electrospray ionization (DESI), the two most widely adopted modalities.^1,2^

Desorption/ionization efficiency can be influenced by a number of factors. These include the physical processes of extraction, ablation, desorption, and ionization, as well as the additional complexity of ionization efficiency of any molecule being governed by the chemical environment of the sample itself.^3^ A challenge in mass spectrometry imaging is uncoupling the variability observed in detected ion intensities, for any detected ion, arising from technical factors from the expected chemical/biological variation between measurements.^3^ The fluctuation in detected ion intensities, which is associated with these “technical factors” and not the variability of molecular composition of the sample itself, may be referred to as “batch effects”.^4^ Technical factors influencing the detected ion intensity are widespread and can be associated with the sample handling and preparation stages, as well as the physical profiling of the sample and the transmission, manipulation, and detection of gas-phase ions within the mass spectrometer.^5^

Mass spectrometry imaging experiments comprise a vast number of discrete measurements (individual pixel-associated mass spectra). Each pixel represents information from a unique location. As a probe-based approach, mass spectra are collected in a sequential manner and consequently are collected at different times (with the exception of microscope mode instrumentation). An MSI data set, or study, can be considered as batches of pixels, samples, and slides. As MSI data are acquired in a sequential manner, with each pixel/spectrum pair being acquired at a unique point of time, it follows that a batch effect may occur within an individual data set. An example of this may be an image gradient, or a signal carryover due to contamination during an experiment.^6^ This is referred to as the pixel-to-pixel batch, and a batch label can be associated with each pixel/spectrum pair. On a higher level exists the experiment–experiment batch effect. Here, all pixel/mass spectra pairs belonging to a given tissue section are assigned as part of the same batch. In order to allow for an assessment of variability, replicate sections of the same biological organ or tissue sample can be included in MS imaging studies.^7^ However, natural biological variations between sections exist, thus potentially compromising the concept of their use as technical replicates. In addition, such natural biological variations may intertwine with other technical variations, further complicating the matter of batch effect correction. This can be mitigated by the use of tissue mimetics, such as frozen and sectioned tissue homogenates or cell pellet materials, which are attractive QC samples and can serve as valid technical replicates.^8,9^ The highest level of batch type may be described at a slide-to-slide level. It is increasingly common for the numbers of sections surveyed in an MSI study to span several slides. In good study designs, each slide might include both technical and biological replicates. On this level, the same batch index may be assigned to multiple sections on a slide. It is at the run-to-run and slide-to-slide levels, where it is most likely that a batch effect may be confounded with an important biological condition. For example, if sections from one biological condition are placed on separate slides, then differences between conditions may be indistinguishable from the slide-to-slide variations. Differences in ionization efficiency, instrument drift, and sample preparation can introduce systematic biases that confound biological interpretations.^3^ Therefore, careful experimental design, which includes practices such as randomization of sample placement must be employed.

Incurring systematic variability, or batch effects, can potentially be addressed by ad hoc or post hoc treatment of the data. Batch correction methods aim to mitigate systematic biases introduced by differences in experimental conditions, instruments, or processing batches in high-dimensional data, such as omics or imaging data sets. In other fields, such as metabolomics and transcriptomics, several batch correction methods have been introduced and commonly adopted.^10^ The approaches can be broadly classified into categories according to the level of supervision needed. Batch correction methods may require the “batch” to be labeled, or they may require the time of acquisition or the individual samples to be labeled. There are various correction algorithms that fall within each category. Some examples that require batch label only include NormAE,^11^ ComBat,^12^ limma,^13^ and MnnCorrect.^14^ On the other hand, some methods use both a batch label and sample type label; for example, ComBat with covariates, limma with covariates, scMerge,^15^ and scGene^16^ all take this approach. Examples of methods that require timestamps of individual acquisitions like BATMAN,^17^ GPfates,^18^ and WaveICA^10^ are used to describe and correct batch effects from a temporal perspective. MSI data are notably different from bulk omics data since there is an additional spatial component. This means that the principles or assumptions of some approaches do not necessarily hold true. For example, WaveICA aims to remove low-frequency variation assumed to be contributing to batch effects but requires a randomized injection order to ensure that biological variation is then in higher frequencies. MSI data acquisition cannot be truly random, however, since neighboring pixels will typically be from the same sample, and QC cannot be randomly introduced throughout the whole imaging run. When the performance of batch correction is evaluated, additional consideration of the preservation of spatial information must also be considered. Potential future technology developments may enable full pixel randomization such as is used in secondary ion mass spectrometry (SIMS) imaging^19^ and inclusion of QC material; however, this is not currently the case in MALDI or DESI MSI.

To date, there is no widely adopted approach to batch correction of mass spectrometry imaging data. Pixel-to-pixel “correction” is actually quite common; for example, mass calibration and spectral alignment procedures are often undertaken to account for a drift in mass accuracy over time. Alternatively, various approaches for quantitation have been applied to MSI, such as quantitation of selected molecules of interest by spraying uniform standards onto samples^20^ or classes of molecules by incorporating standards into extraction solvents.^21^ Other common spectral preprocessing tasks include intensity normalization methods such as the cross-normalization approach introduced by Boskamp et al.^22–24^ The goal of normalization is to scale the intensities of each pixel to remove systematic artifacts that affect intensity. Very recently, Huang et al. compared the use of ComBat, WaveICA, and NormAE for correction of homogenate data acquired across 3 days.^25^ In this work, a gelatin and propranolol-based QC material was used to evaluate correction of three different homogenate materials (chicken heart, chicken liver, and goat liver). Of note in their study, total ion correction alone was sufficient to separate the different sample types by principal component analysis; however, this study only considered mean spectra from the different sample types rather than a pixel-by-pixel approach.

In this paper, we evaluate normalization and batch correction methods for the treatment of MALDI MS imaging data, which were collected over 10 slides and approximately 10 days of analysis. We consider the batch correction approaches evaluated by Huang et al: (1) WaveICA, (2) ComBat, and (3) NormAE, as well as one additional method, SERRF^26^ (a regression model that uses a batch label and a QC sample and uses a random forest to learn the correction) originally intended for lipidomic data. Specifically, in this work, we consider both stabilization of intensity variability and the usefulness of correction methods for spatially resolved data. We present a pixel-by-pixel evaluation of batch correction for mass spectrometry imaging data.

## Methods

### Samples and Sample Preparation

A large study involving a range of different sample types including primary breast cancer (BC) core and large biopsies as well as patient-derived xenografts (PDX) was undertaken. All samples were flash-frozen and randomly thawed and mounted onto 10 different glass slides (SuperFrost Thermo Scientific, Germany). The total study consisted of 121 biological samples. This comprised 10 serial sections of cell pellet material and 10 serial sections of PDX material (the QC samples used within this study). The remaining 101 samples included sections prepared from PDX, patient biopsy, and additional cell pellet material (36 sections of PDX tissues, 62 sections from primary biopsies, and three additional sections from cell pellet material). Serial sections of a cell line (MDA-MB-468) pellet material and a BC PDX (BR1282, Crown Bioscience) were included in each slide as controls. The core biopsy samples were embedded in HPMC/PVP hydrogel embedding media as outlined by Dannhorn et al.,^27^ and all samples were sectioned to 10 μm thickness using a cryostat (Leica Biosystems CM 1950, Germany). The slides were then vacuum-packed and stored at −80 °C, until they were processed for MSI analysis. A 9-amino acridine (9AA) matrix was applied to these samples using a TM-sprayer (HTX Technologies, LLC, Chapel Hill, North Carolina), using 5 mg/mL in 70:30 EtOH/H_2_O at a nozzle temperature of 70 °C using 14 passes at a flow rate of 0.1 mL/min, a velocity of 1200 mm/min, and a 3 mm track spacing. This gave an overall matrix density of 0.001944 mg/mm^2^.

### MALDI

All slides were analyzed consecutively by MALDI MSI using a timsTOF flex instrument (Bruker Corporation, Billerica, Massachusetts), at a 50 × 50 μm pixel pitch in negative ion mode with an *m*/*z* range of 50–1200. These data represent acquisitions taken over the course of 9 days (Figure S2 and Table S1). Instrument calibrations were carried out between scans unless two slides were scanned sequentially while being placed in the same slide holder.

### Data Conversion and Datacube Generation

Bruker’s raw file (.tsf) data were converted and combined into a single imzML file using custom Matlab scripts. Data were initially analyzed with SpectralAnalysis^28^ and Matlab2023b (The MathWorks, Inc., Natick, MA, USA). Spectra were preprocessed using a linear interpolation rebinning (0.0008 Da bin width); following this, a total spectrum was generated. The different tissue pixels were segmented from the background by *k*-means clustering with the similarity metric set to “cosine”^29^ and the number of clusters set to 3. Manual labeling of each tissue type was assigned using the spatial segmentation results and optical images as a reference. Following this, a tissue-specific total spectrum was generated and peaks were picked by the gradient method. A common *x*-axis was created for cell pellet (CP) and PDXs, and the top 1000 peaks were selected for all subsequent analyses for these two types of tissues. A CP-only datacube (17,356 pixels) and a PDX-only datacube (45,254 pixels) were prepared and analyzed by using the above-mentioned batch effect correction methods. In both cases, the top 1000 most intense peaks from CP and PDX joint datacubes were included.

### Batch Correction Methods

Four recent and representative batch effect correction methods, namely, SERRF, WaveICA, ComBat, and NormAE, were selected to analyze our data. Only the original code and language were used whenever possible, as the objective of this paper is merely to test the performance of each method in its native state. This is true for WaveICA, NormAE, and ComBat where the published codes were used. However, SERRF’s R shiny interface can only load input data (in csv format) up to ∼370 MB, which is not suitable for our MSI data. Therefore, we translated SERRF into Python and added an additional functionality to allow users to save the trained model out for correcting additional discrete subject sample data. Versions of all languages and packages were chosen according to the specifications of original papers and source code manuals.

All pixel sampling times, which are usually relative values (in s) with respect to the experiment start time for each slide, were recalculated based on the start time of the first acquired slide and were hence unified into a consistent time array (see Figure S2). This time array will be treated as the injection order array whenever it is required for a method. The temporal order number, from 1 to 10, is coded and used as the batch labels.

#### Normalization

TIC and *g*-log transforms were implemented using the numpy^30^ package. A stabilizing parameter of 10^6^ was used to stabilize the variance for all datacubes. L2 norm and *z*-standardization were performed using the readily available preprocessing modules in the scikit-learn library.^31^

#### SERRF

The original SERRF algorithm^26^ was adapted from https://slfan2013.github.io/SERRF-online/ to enable the handling of data sets larger than 500 MB. In short, 50% of cell pellet pixels were randomly selected as training data, and the remaining 50% retained for evaluation. All pixels were then assigned a batch number, an acquisition timestamp, and a training or testing label. These were then used to train a random forest prediction model to estimate the systematic error and normalize the intensity of each molecule by scaling via this systematic error. The model trained on the cell pellet data was then subsequently applied to the data from the PDX samples. It is not appropriate to train a model directly on the data from the PDX samples as the method assumes a homogeneous QC sample, which is not expected from the PDX samples.

#### WaveICA

The latest version of WaveICA (WaveICA 2.0)^32^ was downloaded from the following location: https://github.com/dengkuistat/WaveICA_2.0, and implemented in RStudio. As with the SERRF method, all pixels were then assigned a batch number and an acquisition timestamp; however, no training and testing split of the data was required.

#### ComBat

ComBat was applied using the Python package neuroCombat.^33^ The parametric version was used with a given batch index corresponding to all of the pixel indices belonging to a particular day. No covariates were used.

#### NormAE

Two input files are needed to run NormAE; these are metabolomics_data and batch_information. Input files were created following the directions provided by Rong et al.^11^ The CP and PDX data sets were coded into 50% QC pixels and 50% sample pixels by a random number generator and used for the training step. Different batch sizes were used for this purpose with the CP data set (64, 320, 640, and 1280), where 1280 provided the best results (i.e., the best PCA score plot and average correlation coefficient). Thus, this batch size was used also for the PDX data set. The batch effect was removed on both data sets with their corresponding output models.

### Postprocessing

#### Violin Plots/Single-Ion Images (SII)

Datacubes were read from a .mat format to numpy arrays using the mat73 Python library. Matplotlib^34^ and seaborn^35^ were used to generate single-ion image plots and violin plots for the different data sets. Single-ion images were generated by using the tissue masks described previously. Violin plots for the classic transform are presented on individual scales. As for the violin plots of the batch-corrected datacubes, the raw data, SERRF, WaveICA, and ComBat were plotted on a shared intensity scale, and NormAE was plotted on an individual intensity scale.

#### Multivariate Analyses (MVA)

Multivariate analyses were performed in Matlab (2017a, MathWorks, USA) using the statistics toolbox. *k*-means clustering was performed using the “kmeans” function with a range of cluster numbers (*k* = 2–10), using the cosine distance metric and 3 replicates.^36^ PCA was performed with the “pca” function with no prior scaling, and t-SNE was performed using the “tsne” function using the exact algorithm and cosine distance metric.

## Results and Discussion

### Multivariate Analysis of Full-Study Data

The data presented here form part of a study on PI3K mutation in breast cancer and consist of a combination of primary core biopsies, patient-derived xenograft tissues, and control samples. Control samples in this instance comprise serial sections of MDA-MB-468 cell line pellets and serial sections of one of the patient-derived xenograft tissues. The arrangement of these tissues on each slide is provided in Figure SI1, and a full-study total ion chromatogram of the cell pellet data is shown Figure SI2.

*k*-means clustering (*k* = 12) was performed on a tiled datacube constructed from all 10 slides, imaged over the course of 10 days. For visualization purposes, the images were tiled such that each tissue type was displayed in a single column, with rows corresponding to different slides (Figure 1). Notably, columns one and two, which depict the segmentation results for the PDX technical repeats and CP control samples, respectively, show a striking consistency across all slides.

**Figure 1:**
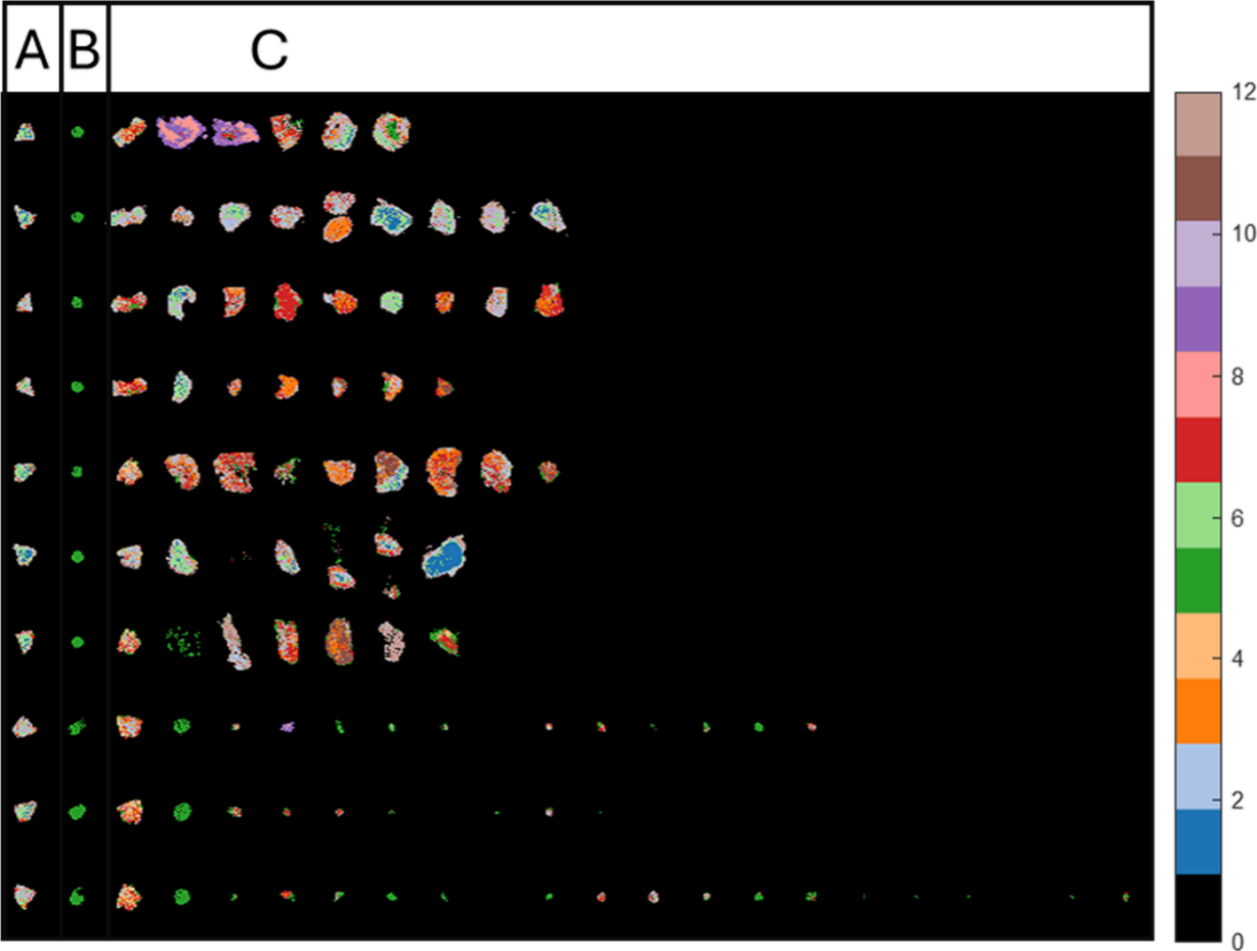
Segmentation results (*k*-means, *k* = 12) for all 10 slides tiled together. The image is tiled such that the same tissues of the same type fall within a single column and tissues from the same slide fall within a single row. Cluster numbers are displayed on the color bar. Positions of the PDX control (A), cell pellet control (B), and all other study samples (C) are shown in the labeled columns. Data presented in each row were acquired from the same slide.

The segmentation results in column one indicates that the PDX samples consist of mostly the same clusters (clusters two and four), with secondary clusters (clusters one, three, and seven) appearing across different slides. The CP sample (column two) was consistently characterized throughout the entire study by a single cluster (number five). At first glance, it may therefore appear that this segmentation successfully captured the major biological differences in the study without interference from batch effects. However, in a large, multipatient, multiorgan, multitissue MSI data set such as this, the manifestation of batch effects in highly similar control samples will potentially exhibit smaller variance than what exists throughout the study overall. Therefore, even if they exhibit significant batch effects, control sample pixels may still cluster together as they are still more similar than, e.g., human tissue biopsy pixels, as we would expect. Therefore, this representation does not allow us to rule out or easily study the presence of batch effects. Therefore, we next examined the control samples in isolation.

In a mass spectrometry imaging study involving many samples, some of which exhibit stark differences, *k*-means clustering may preferentially segment clusters that correspond to these pronounced differences over more subtle ones, such as potential batch effects. This occurs because *k*-means minimize within-cluster variance, making it more likely to assign centroids to clusters with the largest separation. As a result, clusters may disproportionately capture highly distinct molecular distributions, while less pronounced variations within similar samples receive less differentiation. Consequently, the clustering outcome may be biased toward segmenting the most contrasting features rather than providing a balanced representation of all variations present across the data set. In this case, this implies that the CP samples, when compared against the rest of the study, are likely over-represented. It is therefore possible that more subtle biological differences, which may have been further obscured by batch effects, were missed in this analysis. This may also be the case in the recent work presented by Huang et al.,^25^ where the large differences between the spectra from the different homogenate materials might mask more subtle pixel-to-pixel and slide-to-slide changes observed in the QC material alone. To investigate this possibility in our data, we examined the behavior of multivariate analysis (PCA, t-SNE, and *k*-means clustering) on two datacubes: one that includes only the cell pellet sample and another that includes only the PDX technical repeat samples. MVA on the raw cell pellet images, alongside commonly applied correction schemes, namely, TIC normalization, L2 normalization, *z*-score standardization, and *g*-log variance stabilization, describes the slide-to-slide variability missed out on by the simple *k*-means approach that was discussed previously. However, as the cell pellet sections were prepared in an identical manner, the slide-to-slide variability indicates the presence of a batch effect.

### Multivariate Analysis of the Cell Pellet Material

The batch effect can be clearly seen in the images of the first principal component scores for each category. Those are presented at the top in Figure 2a–e, corresponding to raw data, TIC normalization, L2 normalization, *z*-score standardization, and *g*-log variance stabilization. The middle two images in each of the subfigures correspond to *k*-means segmentation with *k* = 2 and 4, and the bottom image is a t-SNE RGB image where each dimension is represented as red, green, and blue.^37^ The t-SNE RGB images also reveal that the cell pellet material exhibits some internal heterogeneity. Generally, depending on the correction scheme chosen, the MVA images offer different perspectives on the batch effect. This is corroborated by the scatter plots for the first two principal component scores displayed in Figure 2f–j), corresponding to raw data, TIC, L2, *z*-score, and *g*-log correction schemes. As evidenced by the drop in variance explained by the first principal component, these corrections aim to enhance the analytical capability of PCA. However, if the dominant source of variation in the data is a systematic batch difference, those corrections can end up making that technical difference the largest remaining variance component. Indeed, when compared to the raw data, the separation between batch point clouds lying on the plane of the first two principal component scores increases after applying any of those correction schemes, suggesting that commonly applied corrections may accentuate a batch effect. Most notably, unlike Huang et al., we found that when considering the QC data alone, and in a pixel-wise manner, TIC normalization performed poorly in mitigating the batch effects observed in these data.

**Figure 2:**
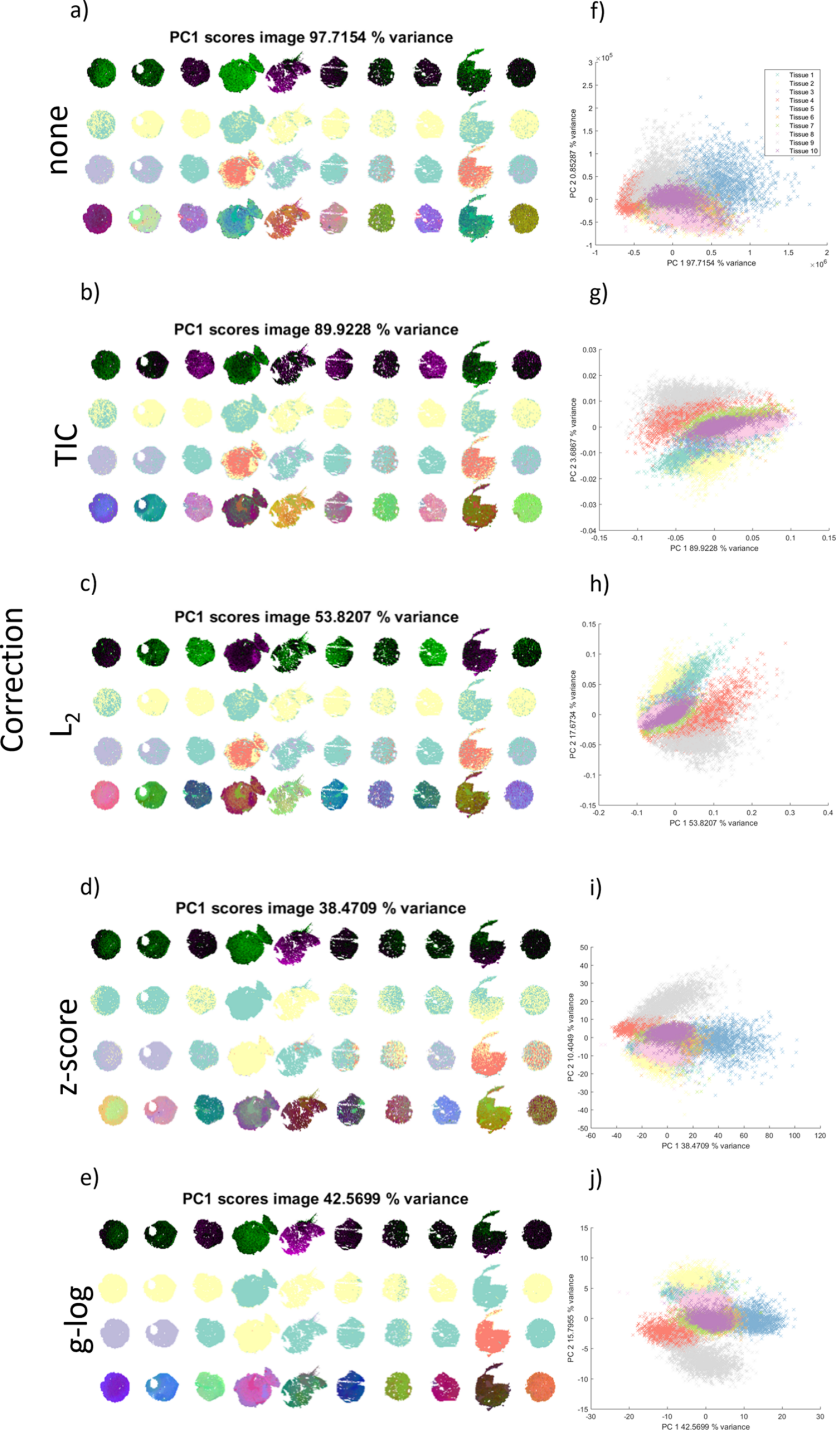
Multivariate view of a batch effect manifested in the control cell pellet data. Data analysis was repeated with five different normalization strategies: none, TIC, L2, *z*-scored standardization, and *g*-log variance stabilization for each row of subfigures as labeled on the left. Cell pellet section images in (a–e) have been labeled, column-wise, by their batch number (one to ten from left to right), corresponding to the chronological order of data acquisition. (a–e) By row: (1) PCA first component score images; *k*-means clustering images, with (2) *k* = 2 and (3) *k* = 4; (4) t-SNE RGB images. (f–j) PCA scatter plots of first two principal component loadings. Five subfigures are shown for different correction types: (a–e) PCA first component score images followed by *k*-means clustering images, with *k* = 2 and 4, and t-SNE RGB images from top to bottom, corresponding to no correction, TIC normalization, L2 normalization, *z*-scored standardization, and *g*-log variance stabilization. (f–j) PCA scatter plot of the first two principal component loadings, corresponding to no correction, TIC normalization, L2 normalization, *z*-scored standardization, and *g*-log variance stabilization (tissue labeling in (f) applies to all scatter plots).

A more detailed view of such variations is thus provided as a review of single ions under the same normalization schemes (Figure 3).

**Figure 3:**
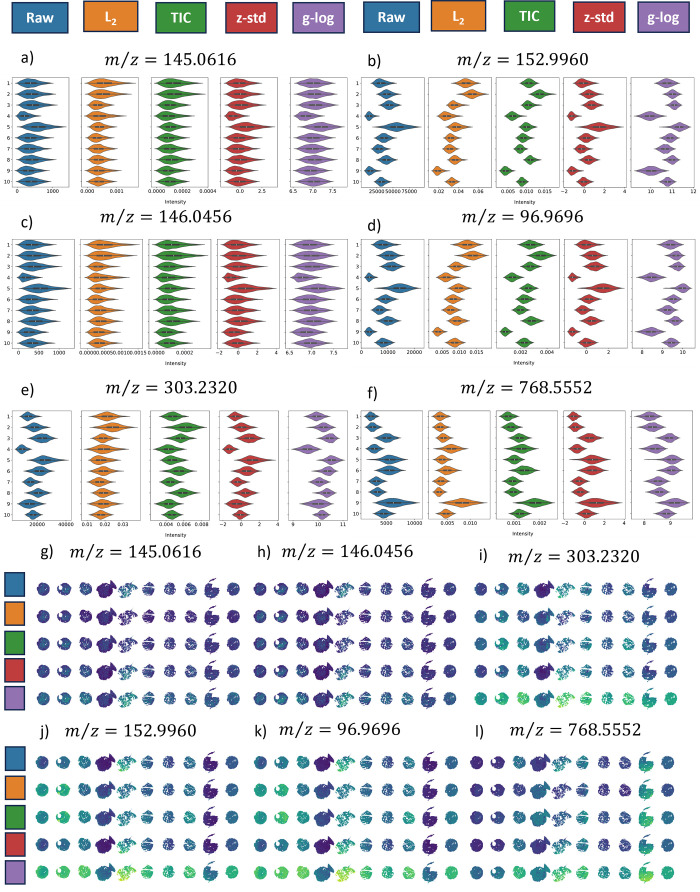
Univariate view of a batch effect manifested in the control cell pellet data. Color coding for each correction scheme is provided at the top of the figure. Violin plots for each correction method and for six ions are shown in (a–f), with their measured mass-to-charge values shown in the titles and chronological batch labels (1–10) on the *y*-axis of each subfigure.

### Univariate Analysis of the Cell Pellet Material

The behavior of six ion signals through the lens of univariate statistics is now provided. These were chosen as exemplar ions revealing a range of observed trends. The ions we have selected span our *m*/*z* range and represent several molecular classes. Violin plots of all detected *m*/z are also provided in the Supporting Information. Assignments, tentative by accurate mass, for the ions presented in Figure 3 are as follows: glutamine [M – H ]^−^, C_5_H_9_N_2_O_3_^–^ *m*/*z* = 145.0616; succinic acid [M + Cl]^−^, C_4_H_6_O_4_Cl^–^ *m*/*z* = 152.9960; glutamate M^–^, C_5_H_9_NO_4_^–^ *m*/*z* = 146.0456; sulfate [M + H]^−^, HSO_4_^–^ *m*/*z* = 96.9600; arachidonic acid [M – H ]^−^, C_20_H_31_O_2_^–^ *m*/*z* = 303.2320; and PE 38:3 [M – H]^−^, C_43_H_79_NO_8_P^–^ *m*/*z* = 768.5552. The behavior of these signals is summarized by the violin plots displayed in Figure 3a–f correspondingly, where the violin shape captures the pixel intensity distributions and the box plot within each violin provides summary statistics on the median (white dot) and interquartile range (IQR, thick black box). Additional violin plots for all ions detected are provided in the Supporting Information. Two key observations emerge from the univariate analysis of the raw data (blue violins in Figure 3a–f). Corresponding single-ion images are also provided in Figure S3.

First, the first and second moments (median and IQR) of the image distributions shift from batch to batch. The behavior of the individual intensity means relative to their grand mean was previously discussed in the context of the correlation matrix for the *z*-standardized datacube, and this is now clearly evident in some of the single-ion image distributions. For example, the intensity medians of succinic acid for batches one and two are not particularly high or low, whereas the intensity median for batch nine is notably low (Figure 3b, blue violins). However, in the case of PE 38:3, batches one and two exhibit a lower-than-average median intensity and batch nine exhibits a boost in signal intensity (Figure 3f). Additionally, the second moments also vary between batches, suggesting fluctuations in instrument variability, as indicated by the expanding and shrinking widths of the violins.

Second, it is evident that the batch effect manifests differently for different ions. If the glutamine and glutamate image distributions were to be used to judge the strength of the batch effect over this study, then it could be concluded that the batch effect is mild, if not negligible, as they both display consistent intensity medians and IQRs across all 10 images. In contrast, succinic acid, sulfate, arachidonic acid, and PE 38:3 show a sharp increase in median intensity in the first three batches, followed by a downward trend, along with wide variations in their IQRs. It should be noted that the L2 and TIC normalization strategies bring the individual image distribution medians closer to the group median for glutamine, glutamate, and arachidonic acid; however, they do not equalize the medians for the other three ions, nor do they standardize the IQRs for all six ions. This is particularly important when considering tissue-wise vs pixel-wise analysis in MSI and indicates that more appropriate batch effect correction schemes need to be introduced.

Now that the mechanisms driving a batch effect have been reviewed with the help of univariate analysis and MVA methods have been used to flag the presence of a batch effect, the focus shifts to four batch effect correction algorithms: SERRF, WaveICA-2, ComBat, and NormAE. A detailed description of the principals and assumptions for each method is provided in the Supporting Information. While PCA, PCA scatter plots, *k*-means clustering, and t-SNE were previously used to characterize what a batch effect may look like and UVA techniques gave a granular overview of how a batch effect affects the image intensity distributions, here, we use those techniques to provide heuristic descriptions of the efficacy of those four batch correction methods. The MVA images shown in Figure 4a–j reveal the effects of the batch correction mechanisms.

**Figure 4:**
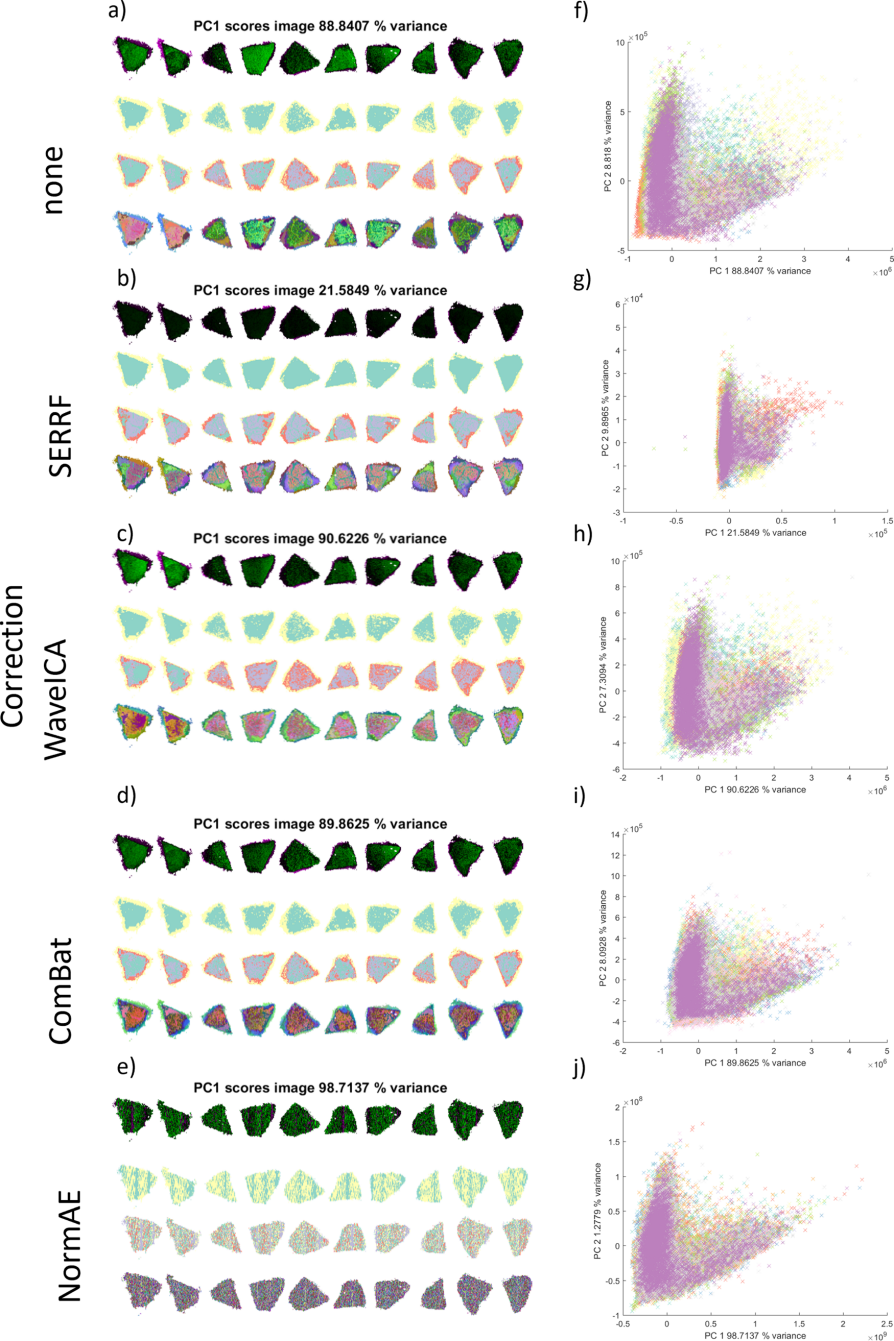
MVA analysis on batch-corrected data for the cell pellet samples' datacube. Cell pellet sections have been labeled by their batch number (one to ten), corresponding to the chronological order of data acquisition. Five subfigures are shown for different correction types: (a–e) PCA first component score images followed by *k*-means clustering images, with *k* = 2 and 4, and t-SNE RGB images; from top to bottom, corresponding to no correction, SERRF, WaveICA, ComBat, and NormAE. (f–j) PCA scatter plots of the first two principal component loadings, corresponding to no correction, SERRF, WaveICA, ComBat, and NormAE.

Notably, most correction methods do not lower the percentage of variance explained by the first principal component compared to the raw data, except for SERRF, where variance explained drops to around 84%. This is because SERRF includes autoscaling steps and because it normalizes the variance associated with correlated peaks. The MVA images convey some information about the performance of the batch correction methods. In particular, both WaveICA and ComBat seem to preserve some of the internal cell pellet heterogeneity (Figure 4c,d), while SERRF- and NormAE-corrected data exhibit no such heterogeneous structures (Figure 4b,e). This can be explained by the assumptions of the different methods. In SERRF and NormAE, the assumption is that the QC sample should be homogeneous, and the goal is to return a data set such that the variance within it is minimized. By contrast, ComBat seeks to align the group means and variance distributions of these samples and to preserve the variance within the sample itself. Similarly, WaveICA seeks to remove low-frequency variation associated with batch effects while preserving high-frequency biological variation. The PCA scatter plots (Figure 4f–j) show that the batches have been successfully aligned, with SERRF exhibiting the tightest point cloud distribution in the space of the first two PC scores.

Additional insight into the performance of each of the batch correction methods is given by the UVA plots shown in Figure 5, where the pixel intensity distributions and their corresponding single-ion images for six tentatively assigned ions and each of the correction methods are presented. Note that the violin plots for the raw data and SERRF, WaveICA, and ComBat batch-corrected data are presented on the same scale, while NormAE is shown on a separate scale due to the fact that NormAE did not always pull images toward their group mean. The NormAE approach uses autoencoders to remove batch effects, and therefore, it is much more challenging to determine and delineate possible sources of this variation. It can be seen that all four batch correction methods successfully align the first and second moments of the single-ion image distributions. Interestingly, SERRF shrinks the second moments for the succinic acid, sulfate, arachidonic acid, and PE 38:3 image distributions but keeps them very similar to those of the raw data for glutamine and glutamate. The principle within SERRF is that the batch effect observed for one ion will describe the batch effect for others. This may be true in metabolomic investigations incorporating chromatographic selection and separation, in which molecules with similar physicochemical properties are surveyed but may not be the case in MSI where no prior separation has occurred. In contrast, NormAE seems to expand the image distributions. As for WaveICA and ComBat, they both keep the shapes of the raw data image distributions intact, indicating that the spatial information in the images is preserved upon correction. The preservation of spatial features after correction by WaveICA indicates that these are higher-frequency changes (distinct features rather than lower-frequency gradients). In the case of ComBat, this preservation is expected as ComBat seeks to align the means and variances of the batches and transforms all pixels within a batch (each cell pellet) equally, thus preserving features within each batch. However, while the cell pellet sections do exhibit some internal heterogeneity, their primary purpose is to serve as a control for batch effects. It would therefore be more informative to review the performance on tissue structures that exhibit a high degree of internal heterogeneity, such as the PDX technical repeats.

### Correction of PDX Tissue Sections

The properties of the four batch effect correction schemes when applied to highly heterogeneous samples are now investigated. For a more succinct analysis, the PC 1 and 2 scatter plots and t-SNE RGB images of the second and third tissues are shown in Figure 6.

Notably, only SERRF and ComBat group together these two tissues and preserve the spatial heterogeneity of the tissue. WaveICA-corrected data still show a large difference between tissues 2 and 3 possibly due to this difference being higher in frequency than it would seek to correct, and NormAE removes all spatial structures in these tissues. A more detailed analysis with all tissues and *k*-means clustering is provided in Figure S5 where panels (a–e) show MVA images (PCA, *k*-means clustering, and t-SNE RGB images), and the PCA scatter plots can be seen in Figure S5f–j. For the PDX samples, a sharp batch effect is observed between the first two samples and the rest, as indicated by the t-SNE RGB image. WaveICA also seems to separate the first two samples from the rest, potentially due to the high frequency of the batch effect itself. As noted, this may be because WaveICA removes low-frequency changes, and the changes between the first two samples and the rest represent high-frequency changes in the data. Importantly, again, NormAE could not recover any spatial heterogeneity in this data set, indicating that a potential overcorrection may have been performed, but as noted earlier, it is challenging to infer the cause of this due to the “black box” nature of the autoencoders. Finally, both SERRF and ComBat appear to successfully harmonize all 10 batches. The fact that ComBat is able to harmonize these batches while WaveICA does not indicates that in this instance, the main difference between batches is the differing means and standard deviations as these are corrected by ComBat. Notably, while SERRF did not preserve spatial information in the data from cell pellets due to its assumption that they were homogeneous, it does preserve spatial information in the PDX samples since they are not part of the training data for the random forests that it implements for correction.

**Figure 5:**
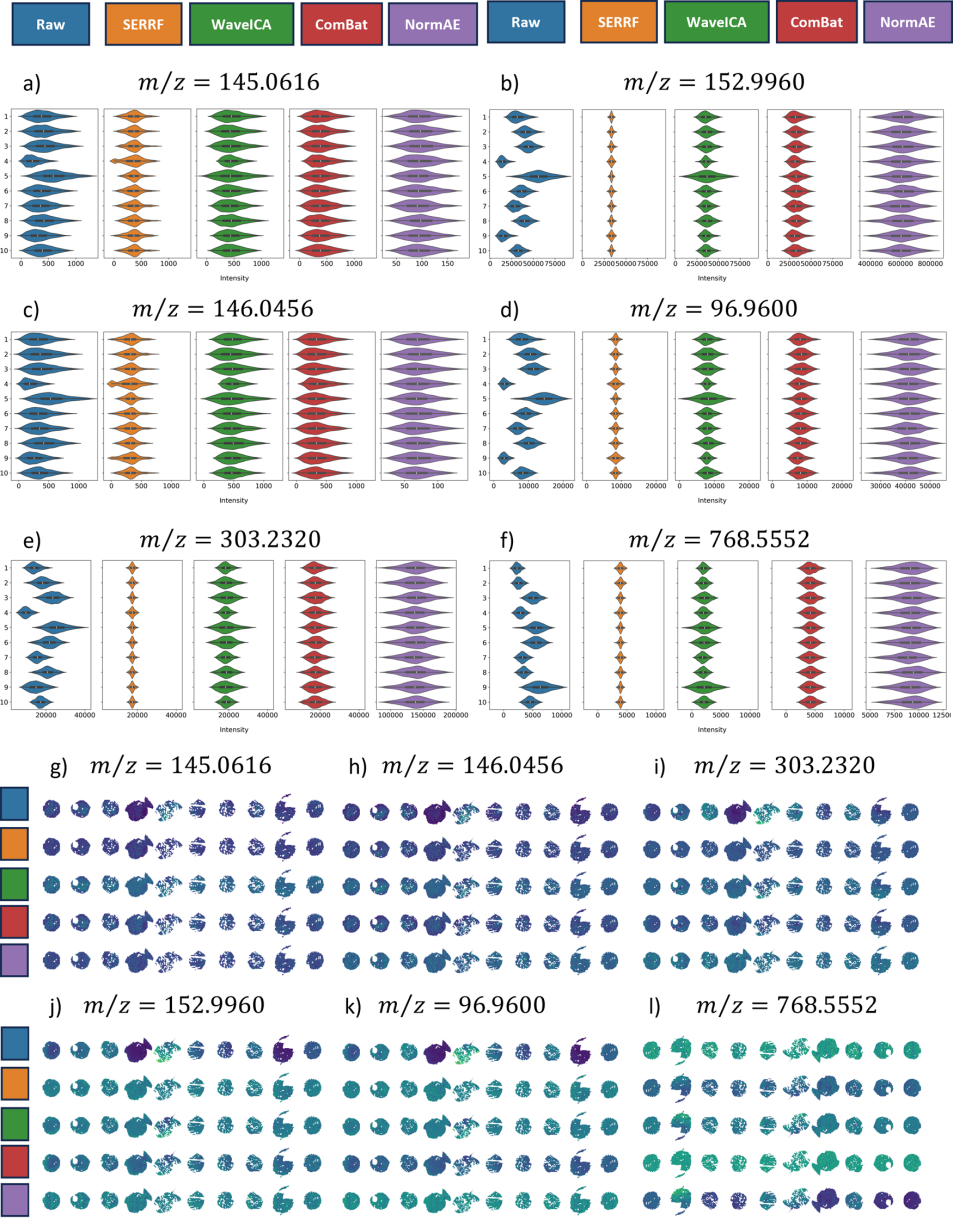
Univariate view of a batch effect manifesting in the control cell pellet samples' datacube. Color coding for each correction scheme is provided at the top of the figure. Violin plots for each correction method and for six ions are shown in (a–f), with their measured mass-to-charge values shown in the titles and chronological batch labels (1–10) from top to bottom. Alongside this are the associated single-ion images with and without correction (g–l). The use of these batch correction methods harmonizes the data between the different batches.

A more in-depth understanding of the behavior of each of those correction schemes can be gained from the UVA descriptions provided in Figures S6 and S7. The violin plots for the six ions of interest provide a rough overview of the performance of each method: SERRF synchronizes the between-batch variations well; however, it maps the grand means to approximately the same constant intensity value; this can be easily seen in Figure S6b, where the grand means of the raw data are plotted against the grand means of SERRF-corrected data, where intensities as high as 10^6^ get mapped to around 10^3^. This behavior is consistent across the entire intensity axis. Nevertheless, SERRF harmonizes the between-batch variances quite well, as seen in Figure S6g, where the SERRF-corrected grand means are plotted against SERRF-corrected between-batch variances; these variances are much lower than the raw between-batch variances, depicted in Figure S6f. Similar to the RAW data, MVA analysis on the WaveICA-corrected data separates the first two batches. The violin plots shown in Figure S5 suggest that this may be due to misalignments in the second moments of individual distributions: the distributions exhibit fairly strong within-batch variations and moderate between-batch variations. Indeed, the WaveICA-corrected grand means are almost mapped to the original raw grand means with moderate misalignments (Figure S6c). However, the between-batch variances (Figure S6h) are not significantly decreased next to the raw data, suggesting that the batch-to-batch variations still remain large. This indicates that these variances are likely to be associated with high-frequency changes, which cannot be mitigated by WaveICA. This is a limitation of using this approach for correction of ion intensities in MSI. In traditional metabolomic experiments, the QC and any other samples can be introduced in a random order, ensuring that their variance remains of high frequency, and subsequent technical variance will be of low frequency. In typical MSI experiments, however, we do not usually acquire the data in random pixel orders (except in the case of some SIMS experiments), and as such, there may be low-frequency biological variability such as molecular gradients in tissues, as well as high-frequency technical variances such as different time gaps between QC samples. ComBat appears to harmonize both the grand means, with intrabatch and interbatch variances quite well, as seen in the violin plots. In addition, ComBat maps the corrected grand means to the raw grand means (Figure S6d) perfectly and lowers the between-batch variances by several orders of magnitude, indicating a significant decrease in batch-to-batch variations. ComBat aligns the average pixel intensity, and so, the spread of pixel intensities aligns more closely across different batches, reducing batch-induced variability without masking biological differences, which is the core principle of the method. NormAE appears to increase the group-corrected means (Figure S6e) and stretch the group-corrected variances (Figure S6j). This stretching can also be observed in some of the violin plots on Figure S5 (e.g., glutamine and glutamate).

**Figure 6:**
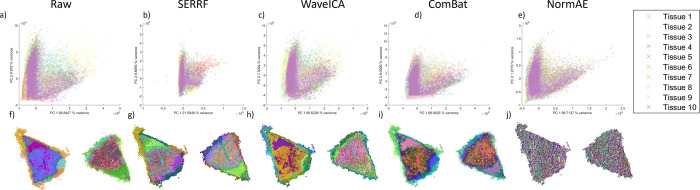
MVA analysis (PCA and t-SNE) of data from selected tissues (slides 2 and 3) of the PDX samples with and without batch correction. The raw data show a small separation between the different tissues by PCA (a), but a clear distinction between the data from slides 2 and 3 is observed by t-SNE (f). The batch correction by all methods improves the PCA overlap (b–e); however, only SERRF and ComBat produce satisfactory t-SNE images (g, i). Correction using the WaveICA method (c, h) produces data that are still differentiated by t-SNE, and NormAE removes all apparent spatial features within the image (j).

This is succinctly summarized in Figure S7, where the batch mean of each group is plotted against its label for the five ions of interest and the four correction methods and the grand mean. For each of those ions, SERRF adjusts them to roughly the same value and minimizes the between-batch variations quite well (Figure S7a–e, orange). WaveICA maps to the grand mean well for sulfate and also reduces the between-batch variations. For similar reasons, NormAE corrects the glutamate ion distributions well. Arguably, it also performs well on succinic acid, sulfate, and arachidonic acid as it reduces the between-batch variation. However, it also significantly deviates from the grand mean. ComBat yields a similar performance to SERRF when reducing the between-batch variations, but it also aligns quite well with the grand mean in all five cases.

### Summary and Reanalysis of the Large Study Data

We can summarize the performance of the different batch effect correction methods according to certain criteria. This can be how well the method corrects batch effects as determined by univariate or multivariate methods and can also be intrinsic properties of the method such as whether separate training data are required or if the method is deterministic. A full summary of the different methods is provided in Table S2. Of note, no perfect method exists yet; however, in the example we have shown, ComBat performs well across almost all criteria we have used.

When seeking to perform batch correction in future MSI studies, a number of considerations must be made: First, it should be noted that while many of the advanced batch correction methods return intensities more closely aligned with the grand mean, there is no guarantee that this is the most appropriate. For example, data sets with low intensity such as poor instrument performance will reduce this mean, and centering may be more appropriate to the grand mean of known “good quality” data sets. Furthermore, some methods such as SERRF, while powerful, require labeled and assumed homogeneous QC samples. These further highlight the need for appropriate QC samples in MSI studies. With this in mind, we have performed batch correction on the data from the entire study presented in Figure 1. In this instance, we selected ComBat to subsequently batch-correct the entire data set earlier described in this study, including the human biopsy data, and then applied *k*-means clustering (*k* = 4, with cosine distance) and PCA to the corrected and uncorrected data. The results from this correction are shown in Figure S8. We can observe in the PC 1 and 2 score plots that in the uncorrected data, a large number of pixels from slide 1 and some from slide 8 are separated from the remaining data in PC 2. After correction using ComBat, these data now overlap with the data from the other slides.

## Conclusions

Initially, we have determined that the measurement and characterization of batch effects in MSI require both univariate and multivariate assessment. We have shown that large-scale MSI studies present relatively unique challenges requiring signal intensity scaling and normalization alongside careful review of expected imaging features and heterogeneity. This scaling and normalization can be different from ion to ion as the degree to which ion intensities appear to be perturbed depends on the ion and slide number. Importantly, without this correction, data from multiple slides cannot be compared alongside each other, potentially invalidating the usefulness of some data sets. In this work, we have reviewed the performance of common batch effect correction algorithms, namely, SERRF, WaveICA, ComBat, and NormAE, as they represent a range of method types. We have shown that the ability to label data according to certain experimental features combined with sophisticated scaling approaches was essential for correction of data over this 10-slide, 10-day MSI study and that for the example shown Combat was the most easily and usefully adopted method. We show that it is also vital to recognize the unique nature of spatially resolved MS data and thus to review the data in a spatial manner, which necessitates a pixel-to-pixel evaluation and correction. Nonetheless, fold changes measured between certain ions and the interpretation of the results would be vastly different without prior correction. We hope that this stimulates further research into the newly emerging area of batch correction for mass spectrometry imaging since further work is needed to develop MSI-specific batch correction methods, which may take into account specific features of MSI instrument performance.

## Supplementary Material





## Notes

Violin plots (ZIP)
Detailed descriptions of the batch correction algorithms, additional figures of the overall study design and layout of the tissue sections, total ion chromatogram of the cell pellet material over the time of the study, additional univariate and multivariate analyses of the cell pellet and PDX samples, and comparison figure of PCA and clustering on the data from the whole study before and after correction via ComBat (PDF)
