# Supplementary material for: Evaluating Batch Correction Methods for Large-Scale Mass Spectrometry Imaging of Heterogeneous Tissues
Source: Anal Chem. 2026 Jan 26;98(5):3531–43. doi: 10.1021/acs.analchem.5c04371 (PMC12903067; doi:10.1021/acs.analchem.5c04371)
Supplement: Supplementary file 2 [file ac5c04371_si_002.pdf]

Supporting information for:

Evaluating batch correction methods for large scale mass spectrometry imaging of heterogeneous tissues

Martin Metodiev [1 †], Alex Dexter[1 †], Weiwei Zhou [2 †], Ariadna González-Fernández [1], Chelsea Nikula[1], Lucy M. Johns[1], Evdoxia Karali[3], Emine Kazanc[3,4], Athanasios Tsalikis[3], Aurelien Tripp[3], Zoltan Takats[5], George Poulogiannis[3], Josephine Bunch [1,5*]

1. National Physical Laboratory, Teddington, UK, TW11 0LW
2. University of Cambridge, Cambridge, UK, CB2 1TN
3. The Institute of Cancer Research, London SW3 6JB, UK
4. Boğaziçi University, 34342 Istanbul, Türkiye
5. Imperial College London, London, W12 0NN

*Correspondence to: josephine.bunch@npl.co.uk

† these authors contributed equally

This file includes:

A detailed description of the different batch correction algorithms

Figures S1 to S8

Table S1 and S2

Full descriptions of the different correction algorithms

QC-based Random Forest Correction Method (SERRF)

QC-based normalisation strategies have been popular in ESI experiments, in which aliquots can be pooled from study samples and injected regularly within batches throughout the experiments. The detected intensities of these QC samples can then be used to establish a regression model describing the systematic error for each detected ion. Assuming the QC sample is sufficiently representative of the target sample, QC-based normalisation methods provide advantage that only unwanted technical variation are to be removed while the underlying biological variation are kept unchanged[1]. The QC-based Random Forest normalisation method, systematic error removal using random forest (SERRF) by Fan et al seeks to extend this, to include the consideration of possible correlations of errors being observed between molecules, rather than only a batch effect or injection order being considered[2]. The fundamental assumption of SERRF is that the systematic variation for each molecule can be better predicted by including the systematic variation of other molecules into the prediction model, which is based on random forest (RF) algorithm.

RF is a machine learning method developed on the basis of combining many single decision trees. RF was chosen for SERRF for a number of advantageous factors that are specifically suitable for mass spectrometry data, where 1) there are more variables (molecules) than samples (sampled data acquisition in MS or pixels in MSI), 2) nonlinear trends often observed in lipidomics[3], 3) high correlation among multiple molecular intensities[4], 4) sizeable missing values (zeros) and outliers[5]. An equally important feature is that RF is known for being able to alleviate overfitting issues by incorporating ensemble of decision trees[6].

Specifically for the *i*th molecule, SERRF constructs a prediction model by including the batch effect *B*, sample acquisition time *t*, and the intensity of QCs from other molecules $I_{-\mathrm{i,\,QC}}$ as the predictor to estimate the systematic error s_i.

$$s_i = \Phi\left(t, B, I_{-\mathrm{i,\,QC}}\right)$$

Equation S1. Prediction model for the SERRF method

and normalise the intensity of each molecule by scaling via the systematic error:

$$I_i' = \frac{I_i}{s_i}\bar{I}_i$$

Equation S2. Normalisation equation for the SERRF method

Where $\bar{I}_i$ is to ensure that the normalised data are comparable to the raw data intensity.

However, SERRF method has only been used for treating lipidomics data, where some considerations cannot be directly extended to mass spectrometry imaging data. Primarily, the biological features were highly reflective in the pooled QCs when running mass spectrometry for lipidomics, whereas for tissue imaging the suitability of the biological features in potential QC has yet to be established. More importantly, the manifested batch effect in tissue imaging can be attributed to the combination of many different possible factors such as instrumental drifting, scanning mode, exposure time of tissue in room temperature, etc.

Wavelet Transformed Based Correction Method (WaveICA)

In a large-scale LC/MS metabolomics data, strong autocorrelation can be found in the time trend presented in peak intensity. Deng et al proposed using wavelet transform (WT) to

decompose such time trend into different level of scales with unique frequencies[7, 8]. Their algorithm, termed WaveICA, effectively separates information affected by different sources of variability. For example, biological differences often exist in higher frequency as different groups of samples are usually randomised, whereas the long-term batch effect exists in lower frequency. The first step of implementing WaveICA is to use maximal overlap discrete wavelet transform (MODWT) method[9, 10] to decompose raw metabolomics data into different frequencies, which will generate approximation coefficients and detail coefficients. From this, the biological effects observed in metabolomics are expected to manifest in the high frequencies(due to randomised injection ordering), and back effects will manifest in low frequencies. Independent component analysis (ICA) is used to threshold and remove batch effect information from each wavelet component[11]. Following deployment of an ICA method, generating independent components, an F-test (or a generalized additive model as in an updated WaveICA 2.0 method) is used to detect and remove any components that are associated with batch effects. Batch effect free data are then reconstructed by using inverse MODWT.

WaveICA may be well suited to MSI studies as it does not rely on any QCs and the time trends in peak intensity could potentially present multiple levels of frequencies due to complexity of image data. However, unlike LC/MS metabolomics data MSI data cannot be acquired in a completely random order due to typical adoption of fixed and ordered raster scanning patterns.

Deep Adversarial Learning Model: Normalization Autoencoder (NormAE)

Inspired by the autoencoder and deep adversarial learning, Rong et al proposed a novel deep learning model called the normalization autoencoder (NormAE) to remove batch effects from metabolomics data[12]. Deep neural networks (DNNs) have been extensively applied to areas such as computer vision[13], natural language processing[14], speech recognition[15], and image analysis[16]. As a type of machine learning models, DNNs consists of many neuron-like processing units and enables them to work in parallel and arranged through interconnected layers. These successive hidden layers can compute increasingly more complex features by considering the outputs of preceding layers. A typical unsupervised learning approach of DNNs is called autoencoder (AE), which aims learn and reconstruct the latent representation of the data through embedding the original high-dimensional data into low-dimensional space via an encoder, followed by transforming them back to high-dimensional space via a decoder. The existence of low-dimensional space, i.e. bottleneck layer, forces DNNs to only extract most representative and important features of the original data.

Based on the fundamental concept of DNNs, NormAE was developed as a type of modified generative adversarial network (GAN) allowing the original mass spectrometry data to be encoded into latent representations with anticipated biological features without the batch effects. The encoder seeks to embed the data into a latent representation that exclude batch effects-related features. The decoder can then reconstruct the data with batch effects removed. Meanwhile, a discriminator is trained to be able to best classify the batch effects based on the latent representations. This approach allows variability associated with biology to be retained.

ComBat

ComBat is a widely used algorithm used to remove non-biological experimental variations. Originally developed for microarray data[17] it has since gained popularity in many bioimaging fields, such as magnetic resonance imaging[18], fluorescence, confocal and immunofluorescence microscopy[19], and computed tomography[20]. While ComBat was not

initially proposed for imaging data, the widespread use of the algorithm across multiple bioimaging disciplines highlights its potential for MSI.

ComBat can be described as a location/scale adjustment correction algorithm and thus addresses both the mean (location) and scale (variance) discrepancies across batches. ComBat aligns the average pixel intensity, and so the spread of pixel intensities align more closely across different batches, reducing batch induced variability without masking biological differences. To achieve this, ComBat employs an empirical Bayes (EB) framework. The EB method first estimates batch specific parameters: the mean and variance from each feature within each batch. It then uses prior distributions and assumes that after standardisation each batch-specific mean and variance follows a global prior trend across all batches. This assumption allows ComBat to shrink individual batch estimates toward the pooled values using EB, balancing the unique characteristics of each single ion batch with the general batch distribution, observed across all *m/z* channels. This shrinkage process is particularly valuable for small batches or noisy data, as it prevents overfitting to individual batch characteristics by regularising estimates towards common trends.

(1) Livera, A. M. D.; Sysi-Aho, M.; Jacob, L.; Gagnon-Bartsch, J. A.; Castillo, S.; Simpson, J. A.; Speed, T. P. Statistical methods for handling unwanted variation in metabolomics data. *Analytical chemistry* **2015**, *87* (7), 3606-3615.
(2) Fan, S.; Kind, T.; Cajka, T.; Hazen, S. L.; Tang, W. W.; Kaddurah-Daouk, R.; Irvin, M. R.; Arnett, D. K.; Barupal, D. K.; Fiehn, O. J. A. c. Systematic error removal using random forest for normalizing large-scale untargeted lipidomics data. **2019**, *91* (5), 3590-3596.
(3) Smolinska, A.; Blanchet, L.; Coulier, L.; Ampt, K. A.; Luider, T.; Hintzen, R. Q.; Wijmenga, S. S.; Buydens, L. M. Interpretation and visualization of non-linear data fusion in kernel space: study on metabolomic characterization of progression of multiple sclerosis. *PLoS One* **2012**, *7* (6), e38163.
(4) Shah, A. D.; Bartlett, J. W.; Carpenter, J.; Nicholas, O.; Hemingway, H. Comparison of random forest and parametric imputation models for imputing missing data using MICE: a CALIBER study. *American journal of epidemiology* **2014**, *179* (6), 764-774.
(5) Rodriguez-Galiano, V. F.; Ghimire, B.; Rogan, J.; Chica-Olmo, M.; Rigol-Sanchez, J. P. An assessment of the effectiveness of a random forest classifier for land-cover classification. *ISPRS journal of photogrammetry and remote sensing* **2012**, *67*, 93-104.
(6) Zhu, T. Analysis on the applicability of the random forest. In *Journal of Physics: Conference Series*, 2020; IOP Publishing: Vol. 1607, p 012123.
(7) Deng, K.; Zhang, F.; Tan, Q.; Huang, Y.; Song, W.; Rong, Z.; Zhu, Z.-J.; Li, K.; Li, Z. J. A. C. A. WaveICA: A novel algorithm to remove batch effects for large-scale untargeted metabolomics data based on wavelet analysis. **2019**, *1061*, 60-69.
(8) Deng, K.; Zhao, F.; Rong, Z.; Cao, L.; Zhang, L.; Li, K.; Hou, Y.; Zhu, Z.-J. WaveICA 2.0: a novel batch effect removal method for untargeted metabolomics data without using batch information. *Metabolomics* **2021**, *17*, 1-8.
(9) Mallat, S. G. A theory for multiresolution signal decomposition: the wavelet representation. *IEEE transactions on pattern analysis and machine intelligence* **2002**, *11* (7), 674-693.
(10) Roushangar, K.; Dolatshahi, M.; Alizadeh, F. MODWT and wavelet coherence-based analysis of groundwater levels changes detection. *Paddy and Water Environment* **2023**, *21* (1), 59-83.
(11) Monakhova, Y. B.; Rutledge, D. N. Independent components analysis (ICA) at the “cocktail-party” in analytical chemistry. *Talanta* **2020**, *208*, 120451.

(12) Rong, Z.; Tan, Q.; Cao, L.; Zhang, L.; Deng, K.; Huang, Y.; Zhu, Z.-J.; Li, Z.; Li, K. NormAE: deep adversarial learning model to remove batch effects in liquid chromatography mass spectrometry-based metabolomics data. *Analytical chemistry* **2020**, *92* (7), 5082-5090.
(13) Esteva, A.; Chou, K.; Yeung, S.; Naik, N.; Madani, A.; Mottaghi, A.; Liu, Y.; Topol, E.; Dean, J.; Socher, R. Deep learning-enabled medical computer vision. *NPJ digital medicine* **2021**, *4* (1), 5.
(14) Otter, D. W.; Medina, J. R.; Kalita, J. K. A survey of the usages of deep learning for natural language processing. *IEEE transactions on neural networks and learning systems* **2020**, *32* (2), 604-624.
(15) Noda, K.; Yamaguchi, Y.; Nakadai, K.; Okuno, H. G.; Ogata, T. Audio-visual speech recognition using deep learning. *Applied intelligence* **2015**, *42* (4), 722-737.
(16) Chen, X.; Wang, X.; Zhang, K.; Fung, K.-M.; Thai, T. C.; Moore, K.; Mannel, R. S.; Liu, H.; Zheng, B.; Qiu, Y. Recent advances and clinical applications of deep learning in medical image analysis. *Medical image analysis* **2022**, *79*, 102444.
(17) Johnson, W. E.; Li, C.; Rabinovic, A. Adjusting batch effects in microarray expression data using empirical Bayes methods. *Biostatistics* **2007**, *8* (1), 118-127.
(18) Fortin, J.-P.; Parker, D.; Tunç, B.; Watanabe, T.; Elliott, M. A.; Ruparel, K.; Roalf, D. R.; Satterthwaite, T. D.; Gur, R. C.; Gur, R. E. Harmonization of multi-site diffusion tensor imaging data. *Neuroimage* **2017**, *161*, 149-170.
(19) Fortin, J.-P.; Cullen, N.; Sheline, Y. I.; Taylor, W. D.; Aselcioglu, I.; Cook, P. A.; Adams, P.; Cooper, C.; Fava, M.; McGrath, P. J. Harmonization of cortical thickness measurements across scanners and sites. *Neuroimage* **2018**, *167*, 104-120.
(20) Arevalo, J.; Su, E.; Ewald, J. D.; Van Dijk, R.; Carpenter, A. E.; Singh, S. Evaluating batch correction methods for image-based cell profiling. *Nature Communications* **2024**, *15* (1), 6516.
(21) Harris, C. R.; McKinley, E. T.; Roland, J. T.; Liu, Q.; Shrubsole, M. J.; Lau, K. S.; Coffey, R. J.; Wrobel, J.; Vandekar, S. N. Quantifying and correcting slide-to-slide variation in multiplexed immunofluorescence images. *Bioinformatics* **2022**, *38* (6), 1700-1707.
(22) Ligero, M.; Jordi-Ollero, O.; Bernatowicz, K.; Garcia-Ruiz, A.; Delgado-Muñoz, E.; Leiva, D.; Mast, R.; Suarez, C.; Sala-Llonch, R.; Calvo, N. Minimizing acquisition-related radiomics variability by image resampling and batch effect correction to allow for large-scale data analysis. *European radiology* **2021**, *31* (3), 1460-1470.

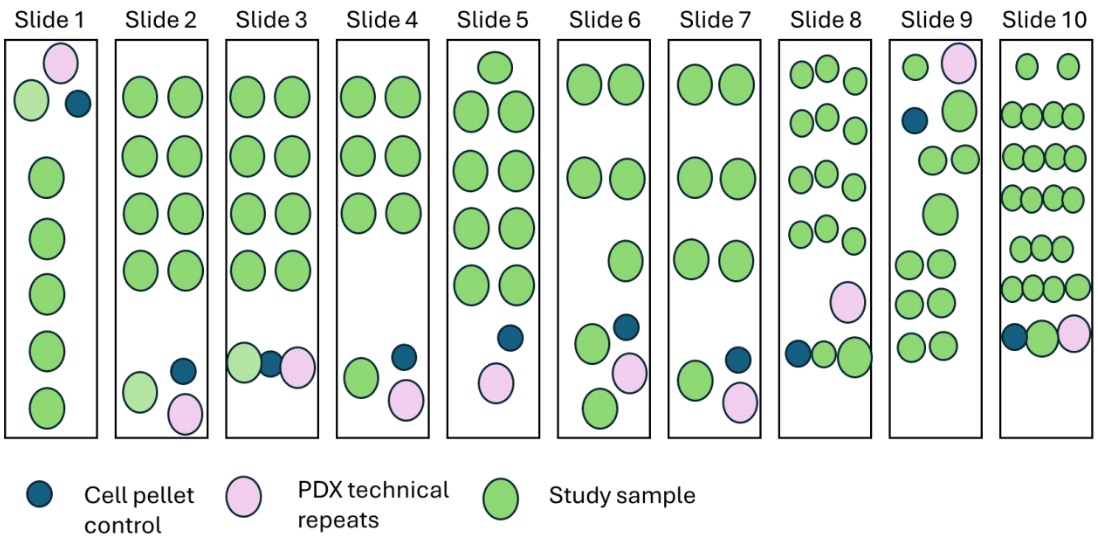

Figure S1. Schematic of the layout of the different tissue samples arranged on each of the ten slides showing the study samples (green), cell pellet control material (blue), and serial sections of PDX tissues (pink).

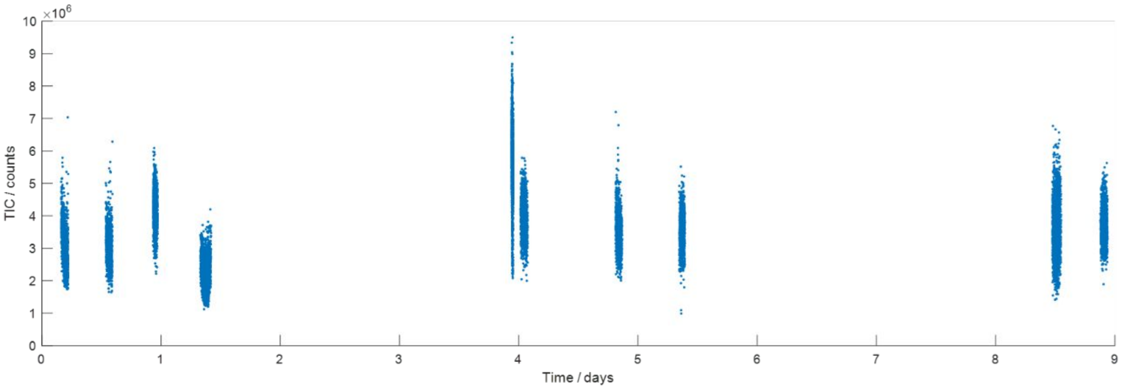

Figure S2. Total ion chromatogram for the spectra from the cell pellet control material acquired throughout the study.

	Start date	Start time	End date	End time
Slide 1	21/04/2022	19:30	22/04/2022	06:31
Slide 2	22/04/2022	06:32	22/04/2022	15:13
Slide 3	22/04/2022	16:24	22/04/2022	21:44
Slide 4	22/04/2022	21:45	23/04/2022	06:19
Slide 5	25/04/2022	13:26	25/04/2022	19:02
Slide 6	25/04/2022	19:03	26/04/2022	02:49
Slide 7	26/04/2022	12:59	27/04/2022	00:32
Slide 8	27/04/2022	00:33	27/04/2022	09:44
Slide 9	29/04/2022	22:34	30/04/2022	13:07
Slide 10	30/04/2022	13:08	01/05/2022	00:14

Table S1. Details on the dates and times of each slide acquisition for the full study data.

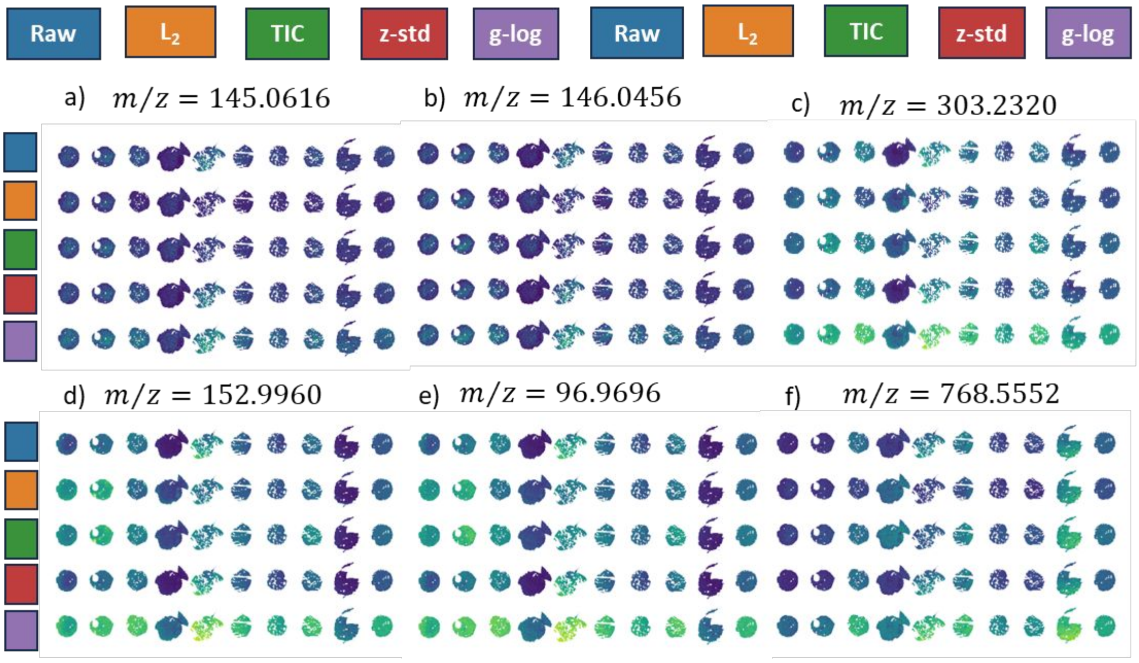

Figure S3. Single ion images for the selected *m/z* signals (a-f) matched to figure 3 with different normalisation methods as per the colour labels. No correction method shown here harmonizes the intensities across all of the cell pellet samples.

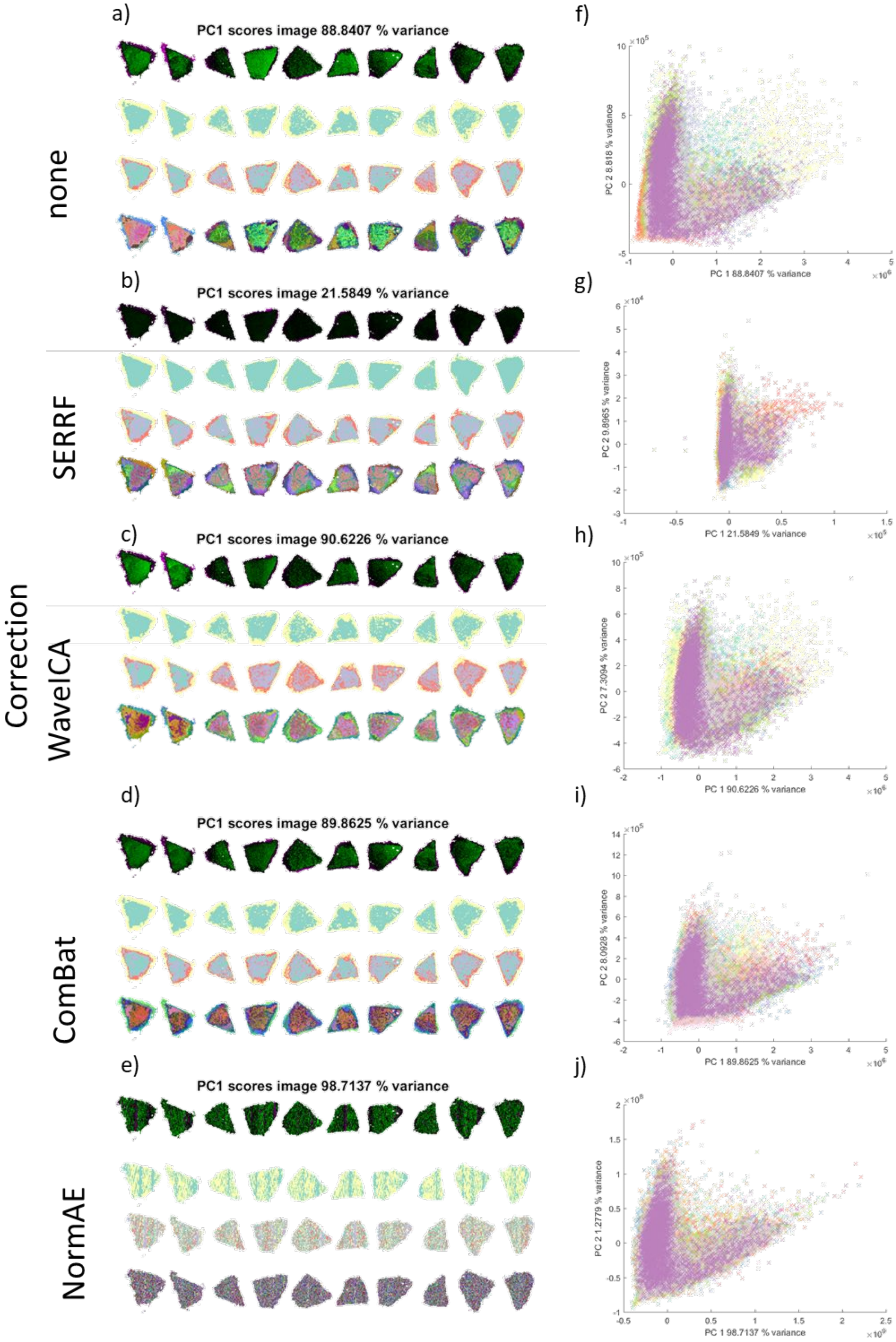
a)
PC1 scores image 88.8407 % variance
b)
PC1 scores image 21.5849 % variance
c)
PC1 scores image 90.6226 % variance
d)
PC1 scores image 89.8625 % variance
e)
PC1 scores image 98.7137 % variance
none
SERRF
WaveICA
ComBat
NormAE
Correction
f)
PC 2 8.818 % variance
PC 1 88.8407 % variance
g)
PC 2 9.8965 % variance
PC 1 21.5849 % variance
h)
PC 2 7.3094 % variance
PC 1 90.6226 % variance
i)
PC 2 8.0928 % variance
PC 1 89.8625 % variance
j)
PC 2 1.2779 % variance
PC 1 98.7137 % variance

Figure S4. MVA analysis on batch corrected data for the PDX datacube. PDX samples have been labelled by their batch number (one to ten), corresponding to the chronological order of data acquisition. Five subfigures are shown for different correction types: a), b), c), d), e) - PCA first component scores image followed by k-means clustering images, with k=2, and 4, and t-SNE RGB image,from top to bottom, corresponding to no correction, SERRF, WaveICA, ComBat, and NormAE respectively. f), g), h), i), j) - PCA scatter plot of first two principal component loadings, corresponding to no correction, SERRF, WaveICA, ComBat, and NormAE.

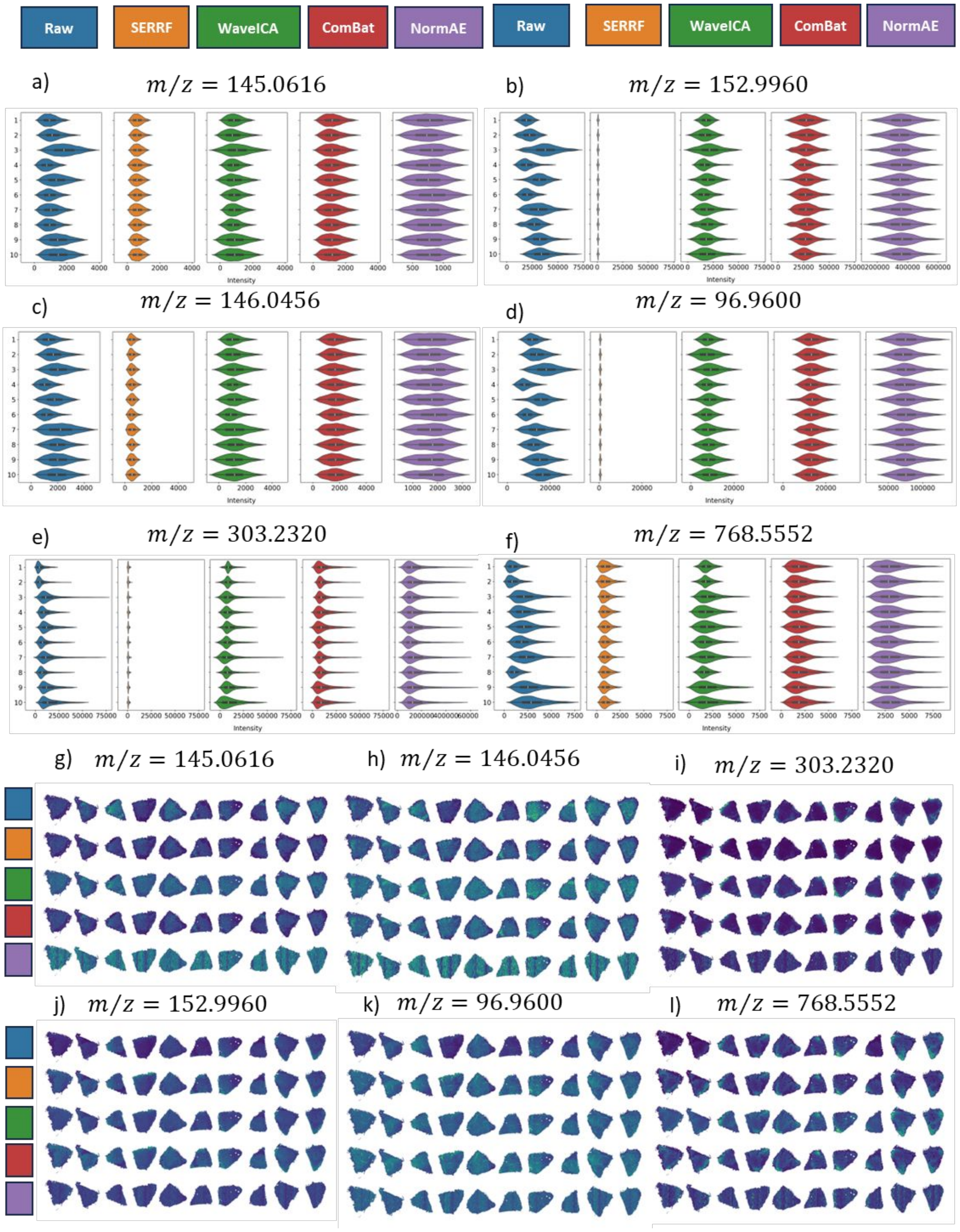

Figure S5. A univariate view of a batch effect manifesting in the PDX datacube. Colour coding for each correction scheme is provided at the top of the figure. Violin plots for each correction method and for six ions are shown in: a), b), c), d), e), f), with their measured mass to charge values shown in the titles and chronological batch labels (1-10) from top to bottom. Alongside this are the associated single ion images with and without correction (g-l). The use of these batch correction methods harmonises the data between the different batches.

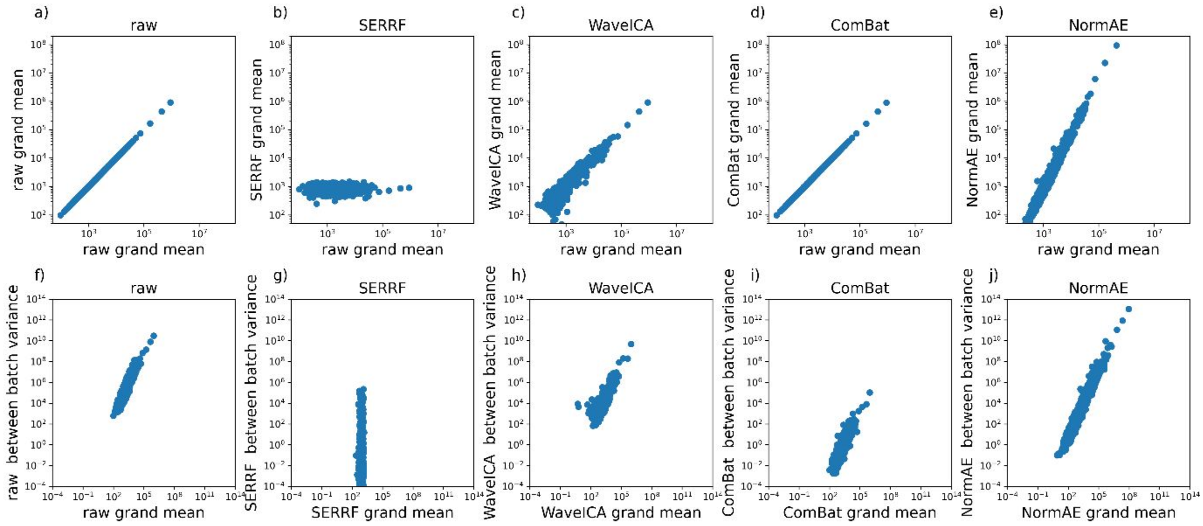

Figure S6. Plots of the raw grand mean against corrected grand mean (a-e), and corrected grand mean against between batch variance (f-j) for the different correction approaches applied to the PDX datacube.

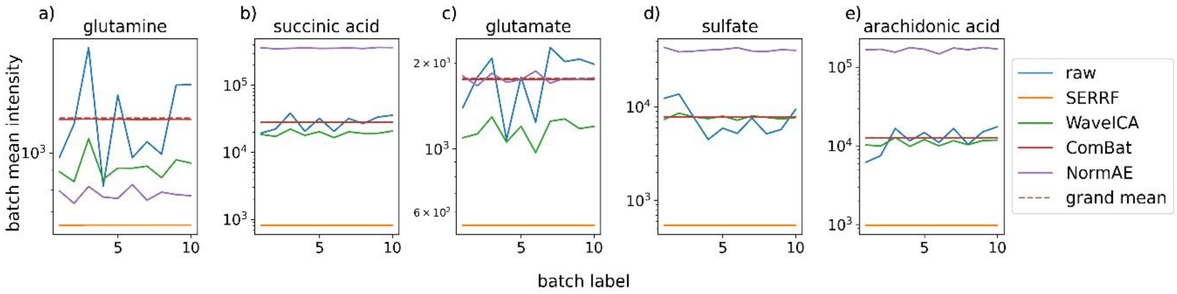

Figure S7. Plot of the batch mean intensity ordered by batch for five different ions described previously (a-e) with the different correction approaches.

	SERRF	WaveICA	NormAE	ComBat
Correct ion intensities between samples	✓	✓	✓	✓
Preserves normality	✗	✓	✓	✓
Retains image structure in heterogeneous samples	✓	✓	✗	✓
Achieve improved overlap by PCA	✓	✓	✓	✓
Reduces the percentage variance explained	✓	✗	✗	✗
Good spectral similarity between batches for cell pellet samples	✓	✓	✓	✓
Good spectral similarity between batches for PDX	✓	✗	✓	✓
Preserves the original intensities	✗	✓	✓	✓
Does not require training data	✗	✓	✓	✓
Model transparency	✓	✓	✗	✓
Deterministic	✓	✓	✗	✓

Table S2 Summary of the key performance and intrinsic characteristics of the different batch correction approaches.

a)

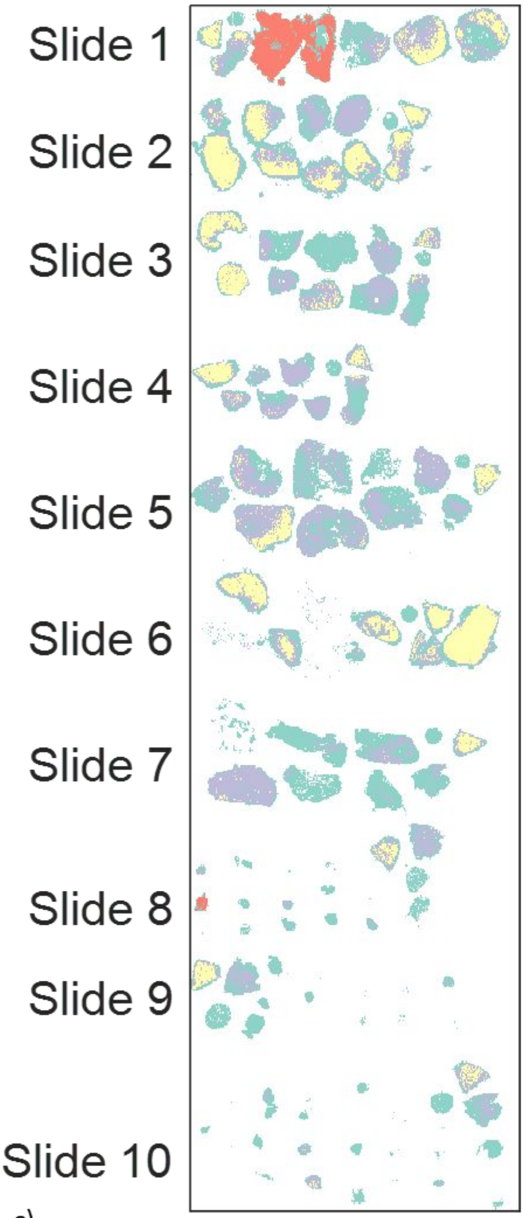

Slide 1
Slide 2
Slide 3
Slide 4
Slide 5
Slide 6
Slide 7
Slide 8
Slide 9
Slide 10

b)

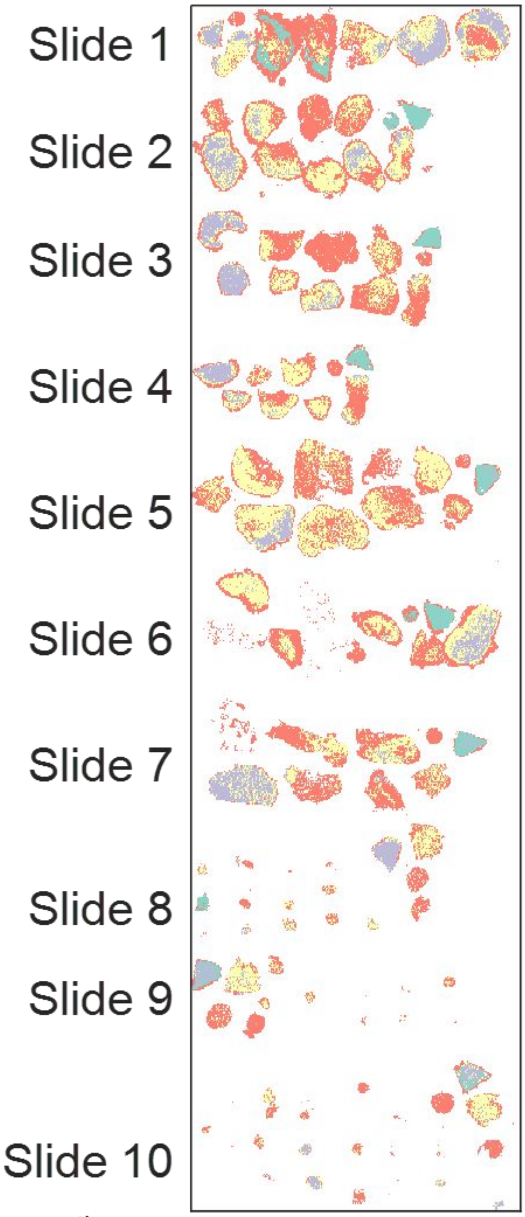

Slide 1
Slide 2
Slide 3
Slide 4
Slide 5
Slide 6
Slide 7
Slide 8
Slide 9
Slide 10

c)

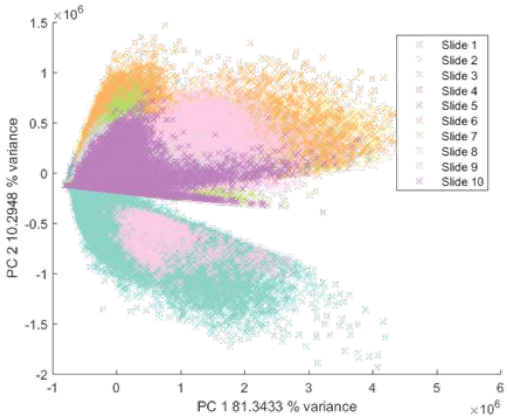

PC 2 10.2948 % variance
PC 1 81.3433 % variance
×10^6
Slide 1
Slide 2
Slide 3
Slide 4
Slide 5
Slide 6
Slide 7
Slide 8
Slide 9
Slide 10

d)

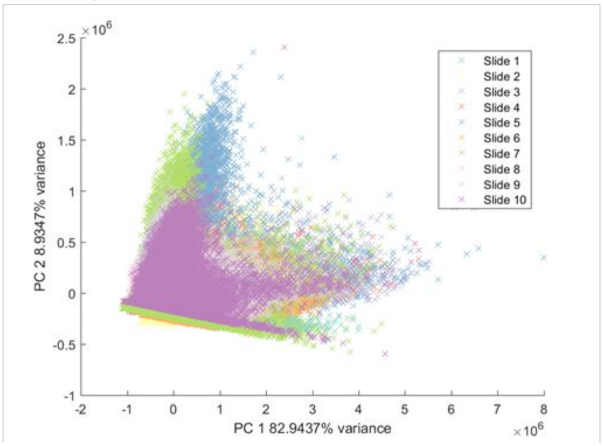

PC 2 8.9347% variance
PC 1 82.9437% variance
×10^6
Slide 1
Slide 2
Slide 3
Slide 4
Slide 5
Slide 6
Slide 7
Slide 8
Slide 9
Slide 10

Figure S8. K-means clustering (a, b), and PCA scatter plot showing PC1 vs. PC2 (c, d) for the data from the entire study before and after ComBat correction. The uncorrected PCA clearly shows a batch effect, with data from slide 1 and 8 separated from the remaining data, which is not observed in the scatter plot after correction.
